# Pan-Genome Analysis Reveals the Abundant Gene Presence/Absence Variations Among Different Varieties of Melon and Their Influence on Traits

**DOI:** 10.3389/fpls.2022.835496

**Published:** 2022-03-25

**Authors:** Yang Sun, Jing Wang, Yan Li, Bin Jiang, Xu Wang, Wen-Hui Xu, Yu-Qing Wang, Pei-Tao Zhang, Yong-Jun Zhang, Xiang-Dong Kong

**Affiliations:** ^1^Key Laboratory for Conservation and Use of Important Biological Resources of Anhui Province, Anhui Provincial Key Laboratory of Molecular Enzymology and Mechanism of Major Diseases, College of Life Sciences, Anhui Normal University, Wuhu, China; ^2^Laboratory for Biology of Plant Diseases and Insect Pests, Institute of Plant Protection, Chinese Academy of Agricultural Sciences, Beijing, China; ^3^Jiguang Gene Biotechnology Co., Ltd., Nanjing, China

**Keywords:** domestication, improvement, pan-genome, presence/absence variation (PAV), resistance gene analogs (RGAs), GWAS, melon

## Abstract

Melon (*Cucumismelo L.*) is an important vegetable crop that has been subjected to domestication and improvement. Several varieties of melons with diverse phenotypes have been produced. In this study, we constructed a melon pan-genome based on 297 accessions comprising 168 Mb novel sequences and 4,325 novel genes. Based on the results, there were abundant genetic variations among different melon groups, including 364 unfavorable genes in the IMP_A vs. LDR_A group, 46 favorable genes, and 295 unfavorable genes in the IMP_M vs. LDR_M group. The distribution of 709 resistance gene analogs (RGAs) was also characterized across 297 melon lines, of which 603 were core genes. Further, 106 genes were found to be variable, 55 of which were absent in the reference melon genome. Using gene presence/absence variation (PAV)-based genome-wide association analysis (GWAS), 13 gene PAVs associated with fruit length, fruit shape, and fruit width were identified, four of which were located in pan-genome additional contigs.

## Introduction

Melon, a diploid, dicotyledonous plant belonging to the Cucumber genus of the Cucurbitaceae family, is an industrial and horticultural crop that is widely cultivated worldwide. At present, China is the largest cultivator of melon. Melon has rich variation types, including plant architecture, fruit size, and color, among others (Stepansky et al., 1999; Mliki et al., 2001). Cultivars are derived from the domestication of wild-type melons, and artificial selection leads to changes in their yield, color, flavor, and other traits, which differ from those of the wild types (Pitrat, 2013). There are two subspecies and 16 different horticultural groups of melon (Jung et al., 2020). Melon varieties originating from China are divided into *ssp. agrestis* (containing var. *conomon* and *var. chinensis*) and *ssp. melo* (containing *var. chadalak*, *var. ameri*, and *var. inodorus*; Giovannoni, 2007). Garcia-Mas et al. (2012) released the reference genome of melon (DHL92) in 2012, thereby laying a solid foundation for melon improvement and subsequent research on the evolutionary origin of melon.

Pan-genome contains the core genes shared by all accessions of a species, as well as the variable genes found in some or several individuals. To a greater extent, the pan-genome represents the genomic variation in one species gene pool. Variable genes help to generate diversity traits of different varieties and may provide advantageous traits that can enable environmental adaptation under certain conditions, such as traits important to yield or response to stress. With the recent development of sequencing technology, the genomes of a large number of individuals have been sequenced. To date, most of the current sequencing focuses on short sequences, and the analysis of variations, including single nucleotide polymorphisms (SNPs), is generally based on the sequence reads mapped to the reference genome to further study the relationship between the evolution or variation of the species and the phenotype (Guo et al., 2021; Wei et al., 2021). However, from the perspective of the pan-genome, these single nucleotide or indel polymorphisms cannot represent the rich variation information between different individuals, especially the presence/absence variation (PAV) and copy number variation (CNV). To comprehensively study the variation of the genome and the changes in important traits induced by gene variation, a pan-genome of a species must be established (Bayer et al., 2020). In the study of tomato pan-genome, 725 representative wild and improved accessions were used to assemble 351 Mb non-reference sequences, resulting in 4,873 new genes, including 74% core genes (present in all accessions) and 26% variable genes (present in at least one accession; Gao et al., 2019). Song et al. (2020) found that by assembling high-quality genomes of eight different varieties of *Brassica napus*, the variation in PAV can reach up to one-sixth of the genome. The size of the *Brassica napus* ZS11 genome is 1 Gb, while the pan-genome constructed from eight high-quality genomes was 1.8 Gb and contained approximately 150,000 genes, of which 56% were core genes, 42% were variable genes, and 2% were cloud genes. Pan-genome analysis revealed that the size of the pan-genome of sorghum is 954.8 Mb, which is 30% larger than the published reference genome of sorghum (BTx623, 732.2 Mb). The length of core gene sequences accounts for 62%, and the length of variable gene sequences accounts for 38% (Tao et al., 2021). Through genome-wide association analysis (GWAS) of sorghum seed color, the Yellow seed1 gene was found to contain an SNP that controls the color of the seed. Combined with the pan-genome of sorghum, a PAV of 3,216 bp was identified. These PAVs have a functional impact on gene structure, thereby affecting agronomic traits. These studies showed that PAVs are popular in plants and play an important role in the genetic determination of phenotypic variation and reveal favorable genotypes during crop improvement.

Plants mainly protect themselves from pathogens through two layers of the biochemical immune system. The first layer is implemented by the cell surface pattern recognition receptor (PRRs), which detects the general inducer pathogen/microbe-associated molecular pattern (PAMP/MAMPs), called PAMP triggered immunity (PTI; Macho and Zipfel, 2015). Plants have also evolved other types of receptors, called resistance (R) proteins, which recognize specific effectors and trigger a powerful counterattack system called effector-triggered immunity (ETI; Jones and Dangl, 2006). Resistance gene analogs (RGAs) include PRR and R genes, and most of them have conserved domains and motifs (Sekhwal et al., 2015). Most of the characterized PRR are surface-localized receptor-like protein kinase (RLK) or membrane-associated receptor-like protein (RLP; Monaghan and Zipfel, 2012; Böhm et al., 2014; Zipfel, 2014). The PRRs can be classified into two groups: surface-localized RLKs (Walker, 1994) and membrane-associated RLPs. RLKs and RLPs are necessary for plant disease resistance (Kruijt et al., 2005). The main category of R gene is composed of nucleotide binding domain (NB) and leucine-rich repeat (LRR) domain, which is usually called (NB-LRR) R gene or NLR (Arora et al., 2019). These plant resistance genes play specific roles in pathogen resistance.

Liu et al. (2020) obtained 2,045,412 SNPs by resequencing 297 melon accessions and analyzing their SNPs, ultimately revealing key signal site selection events in the evolution and improvement of melon fruit traits. In this study, resequencing data of 297 accessions of melons were used for *de novo* assembly, and 168 Mb new sequences were obtained and compared with the reference genome. A total of 4,325 new genes were identified by annotation of the new sequences. Further, the resistance genes of melons were analyzed because of their importance in the process of breeding. Currently, 654 RGA genes have been identified in the reference genome. RGA on the pan-genome was identified and 55 RGA candidates were obtained, which further enriched the research on the resistance gene resources of melons. Using gene PAV-based GWAS analysis, a gene that encodes glutamate receptor 3 was identified to be associated with fruit length.

## Materials and Methods

### Construction of the Pan-Genome

Resequencing data of 297 melon accessions was downloaded from the NCBI Sequence Read Archive (SRA) stored under accession number, SRP192912 (Liu et al., 2020). Fastq-dump of SRA Toolkit^1^ was used to convert the downloaded SRA file to FASTQ format file. Fastp was used to remove adapters and low-quality sequences from raw data.

Similar to the study by Gao et al. (2019), clean data were *de novo* assembled using Megahit with default parameters (Liu et al., 2015). The assembled contigs shorter than 500 bp were removed, and the remaining contigs were aligned with the reference genome and organelle genome of melon using nucmer in the Mummer software package (Marçais et al., 2018). The melon reference genome, DHL92 V3.5.1 (Garcia-Mas et al., 2012), and annotation files were downloaded from the website.^2^ The novel assembled contigs aligned with the melon reference, with sequence identity higher than 90% and longer than 300 bp continually defined as reliable alignments. Contigs with no reliable alignments were defined as fully unaligned contigs. For contigs containing reliable alignments, the sequences were defined as partially unaligned sequences if they contained continuous unaligned regions longer than 500 bp.

After merging the sequences of fully unaligned contigs and partially unaligned sequences, cd-hit-est (Fu et al., 2012) was used to remove redundant sequences. To further remove redundancies, we used blastn and numcer to perform all vs. all alignments, and the in-house perl script was used to compare the results. About 90% sequence identity was set as the threshold value. The final non-redundant sequence was aligned to the nt database using blastn, and the sequences that did not belong to Eukaryota and Viridiplantae were determined using the information obtained in the comparison result. Finally, to ensure that these new sequences did not exist in the reference genome, the sequences obtained in the previous step were aligned with the reference genome using blastn, and similar sequences were filtered using nucmer with the same criteria described above. The final new sequences and the melon reference genome were merged into the pan-genome.

### Pan-Genome Annotation

First, a *de novo* repeat library was constructed using RepeatModeler in the non-reference genome (Flynn et al., 2020). RepeatMasker was used to identify repeat regions of the non-reference genome (Chen, 2004). Tandem repeats were also annotated by the tandem repeats finder (TRF) using default parameters (Benson, 1999). RepeatProteinMask (an application within the RepeatMasker package) was used to identify TEs based on the TE-encoded protein database.

After masking the repeat sequences in the new sequence, we used the maker2 software to predict the gene structure of the genome (Holt and Yandell, 2011). Augustus was used for *de novo* prediction, and melon was used as a model which was trained with annotated files of the melon reference genome (Stanke et al., 2006). The RNA-seq data of 166 accessions were used as evidence for transcription (Supplementary Table 1). For the original RNA-seq data, we used fastp to remove adaptors and low-quality sequences (Chen et al., 2018). Hisat2 was used to align reads to non-reference sequences, and samtools were used to extract reads that mapped to non-reference sequences (Kim et al., 2019). We used trinity to *de novo* assemble the RNA-seq reads mapped to non-reference sequences in each sample (Grabherr et al., 2011). After merging the assembled transcript sequences, cd-hit-est was used to remove redundancies (using default parameters; Fu et al., 2012). Finally, annotations of non-reference sequences were obtained using maker2. By comparing the annotations of non-reference sequences with the repeat sequences, we removed the genes that the overlap of the gene region and the repetitive region was more than 50%. We also used Interproscan (Jones et al., 2014) to annotate the genes in non-reference sequences, and the genes containing the interpro domain were retained. The predicted gene sequences were aligned to the NT, NR, UniProt, and SwissProt databases using BLASTN and BLASTX.

### Identification and Analysis of Gene PAV

All reference genome and non-reference sequences were merged into the melon pan-genome. Bwa was used to align the 297 resequencing data to the pan-genome. We used the ccov in HUPAN (Duan et al., 2019) to calculate the ratio of each gene and CDS region covered by the reads. A gene was considered to exist in a sample if the gene region was covered by the reads of a sample by more than 80%. In contrast, a gene region covered by a sample of less than 80% reads did not exist in this sample. As reported by Gao et al. (2019), genes that exist in all accessions were defined as core genes, genes that exist in 99%–100% of accessions were defined as soft core genes, and genes that exist in 1%–99% of accessions were defined as shell genes; otherwise, genes that exist in less than 1% of accessions were defined as cloud genes.

To identify the gene PAV selected in the process of domestication, we counted the frequency of each gene in landrace *ssp. melo* (LDR_M), landrace *ssp. agrestis* (LDR_A), improved *ssp. melo* (IMP_M), improved *ssp. agrestis* (IMP_A), and wild type (WT). Fisher’s exact test was used to test the significance of the difference in gene frequency between each group, and FDR was obtained from the corrected value of *p* using the Benjamini and Hochberg method. For each comparison, genes with significantly different frequencies (frequency fold change > 2 and FDR < 0.001) between the two groups were identified as selected genes. Furthermore, GO and KEGG enrichment analyses were performed among the selected genes using Fisher’s exact test.

### PAV-GWAS Analysis

The phenotypes (length, width, and shape) of melon were retrieved from Supplementary Table 16 in the paper by Liu et al. (2020). The shell gene was analyzed by PAV-GWAS, and the presence and absence of the gene were used as the genotypes. GWAS analysis was performed using FarmCPU (default parameters) in rMVP (Yin et al., 2021), and the significance threshold was set to 6.4e−6 (0.05/7789).

### Identification and Analysis of Resistance Genes

The RGAugury pipeline (Li et al., 2016) was used to identify resistance gene analogs (RGAs) in the melon pan-genome. RGA includes nucleotide binding site-leucine-rich repeat (NBS-LRR), RLK, RLP, and transmembrane coiled-coil domain protein (TM-CC) candidate genes, which can be divided into 12 subfamilies. These resistance genes were divided into two groups (core and variable genes) based on the results of PAV analysis.

### Identification and Analysis of the TPS Gene Family

The sequences of the AtTPSs were downloaded from the TAIR database.^3^ The terpene synthase-specific domains, PF01397 (terpene synthase N-terminal domain) and PF03936 (metal ion binding domain), were downloaded from the Pfam database (http://pfam.xfam.org/; El-Gebali et al., 2019). HMMER search was performed with HMMER 3.3.2 to search for the Terpene synthase (TPS) gene in melon pan-genome (Finn et al., 2011). The phylogenetic tree was constructed using the neighbor-joining (NJ) method and the Jones–Taylor–Thornton (JTT) model with 1,000 bootstrap replicates in FastTree v2.1.11 (Price et al., 2009). The final phylogenetic tree was redrawn using Figtree version 1.4.4.^4^

## Results

### Pan-Genome Construction and Annotation

We downloaded 297 melon accessions resequencing data from the paper by Liu et al. (2020) in NCBI, including 14 wild-type accessions, 109 IMP_A (improved *ssp. agrestis*), 54 IMP_M (improved *ssp. melo*), and 50 LDR_A (landrace *ssp. agrestis*), 41 copies of LDR_M (landrace *ssp. melo*), 27 ND, and 2 Putative_Feral. According to the evaluation of Gao et al. (2019) for tomato, low-depth data distributed uniformly across the genome can be effectively used for tomato pan-genome research, and data higher than ×4 were preferable in the PAV analysis. The sequencing depth of data used in the present study was more than ×5 on average and was used for the melon pan-genome study.

After merging the fully unaligned contigs and partially unaligned contigs sequences, cd-hit was used to remove redundancy, resulting in 347.63 M sequences. By removing the non-Eukaryota and non-Viridiplantae sequences and performing multiple rounds of all vs. all alignments to remove redundancy, 168 M clean non-reference sequences were obtained.

A total of 4,325 protein-coding genes (PC genes) were predicted in the non-reference genome. The melon reference genome contained 29,980 PC genes. These new sequences and reference genomes were merged into melon pan-genome.

### Gene PAV Analysis

**Figure 1:**
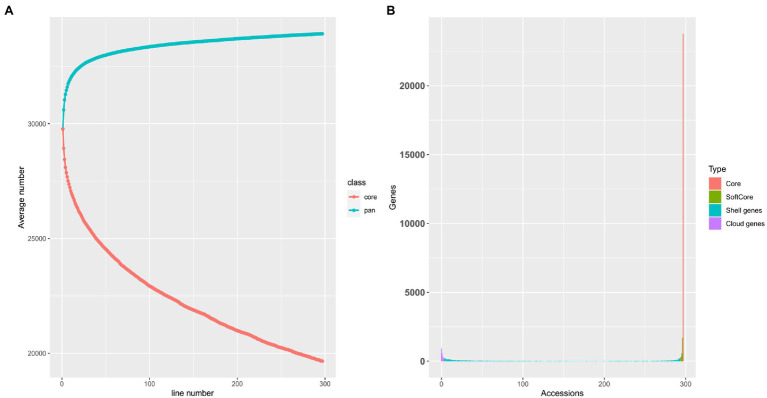
**(A)** The pan-genome modeling shows no new gene increases when the number of accession genomes is greater than 200, indicating that selected individuals were sufficient to capture the majority of presence/absence variations (PAVs) within melon. Upper and lower lines represent the total gene number of pan-genome and core-genome number, respectively. **(B)** Composition of the melon pan-genome.

By calculating the read coverage of each gene in the melon pan-genome, we determined whether the gene exists in each sample. According to the classification criteria described by Gao et al. (2019), a total of 25,200 core and soft core genes, 7,788 shell genes, and 1,317 cloud genes were identified (Figure 1B). Core and softcore genes are relatively conserved in different landraces, improved and wild-type accessions, while shell and cloud genes markedly vary in different accessions. Through an iterative random sampling of pan-genome accessions, the growth rate of the gene number in the melon pan-genome slowed down with an increase in accessions. Based on Figure 1A, when the number of accessions is more than 200, the number of pan-genome genes increased slightly. Furthermore, with the increase in accessions, the number of core genes in the pan-genome decreased, and the core genes presented a significant downward trend when the number of accessions reached 297 indicating that in addition to the selection of natural and artificial genes, genes existing in different accessions were under a stronger selection pressure.

**Figure 2:**
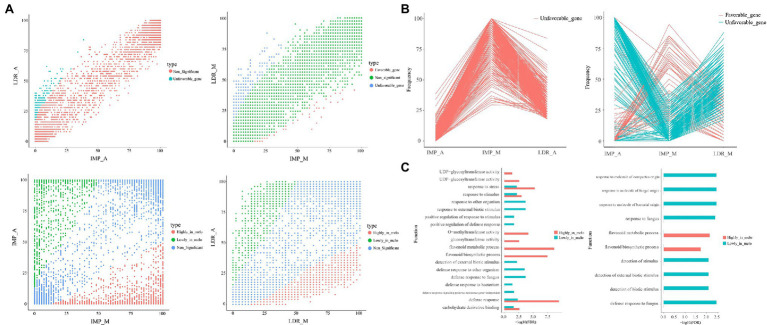
The difference in the frequencies of the selected genes. **(A)** Scatter plots showing gene occurrence frequencies in IMP_A, LDR_A, IMP_M, and LDR_M. The comparison groups include IMP_A vs. LDR_A, IMP_M vs. LDR_M, IMP_M vs. LDR_A, and LDR_M vs. LDR_A. **(B)** Occurrence frequency patterns of favorable and unfavorable genes during improvement. **(C)** Enriched GO terms (FDR < 0.05) in the selected genes of IMP_M vs. IMP_A (left) and IMP_A vs. LDR_A (right).

To identify PAV-related gene under selection during the history of *Cucumis melo* breeding, we performed two sets of comparisons of variable gene frequencies between IMP (containing two subgroups IMP_A and IMP_M) for “improvement” and LDR (containing two subgroups LDR_A and LDR_M) for “domestication.” The comparisons were IMP_A vs. LDR_A and IMP_M vs. LDR_ M. Comparisons between *ssp. melo* and *ssp. agrestis* were performed (IMP_M vs. IMP_A and LDR_M vs. LDR_A; Figure 2A). A total of 14, 27, and two accessions found in WT, ND, and Putative Feral, the number of which was markedly lower than that of other groups, were excluded from the downstream analyses. For each comparison, genes with significantly different frequencies between the two groups were identified as selected genes. We considered genes as favorable genes if they had higher frequencies in IMP than LDR. In the ssp. melo vs. ssp. agrestis group, genes with high frequencies in ssp. Melo than ssp. agrestis were identified as “highly in melo.” In contrast, genes with lower frequencies in IMP than LDR were regarded as unfavorable genes, or genes with low frequencies in ssp. Melo than ssp. agrestis were identified as “lowly in melo.”

No favorable genes were found; however, 364 unfavorable genes were identified in the IMP_A vs. LDR_A group. Further, 46 favorable genes and 295 unfavorable genes were found in the IMP_M and LDR_M groups. By comparing *ssp. Melo* and *ssp. agrestis*, 1,455 and 1,028 favorable genes, and 1,072 and 695 unfavorable genes were identified in the IMP_M vs. IMP_A and LDR_M vs. LDR_A, respectively. These results suggest that more genes tend to be lost rather than gained during melon improvement. Furthermore, the frequencies of the selected genes in the *ssp. melo* and *ssp. agrestis* groups were markedly higher than those in the improvement and domestication groups. Such finding indicates that the difference between the two subgroups was greater than the difference produced by the domestication process. For genes favorable or unfavorable in one subgroup, their trends were roughly the same as those in the other subgroup (Figure 2B), suggesting the possibility of common and continued selection preferences from domestication to improvement.

For the selected genes in the IMP_A vs. LDR_A, IMP_M vs. LDR_M, IMP_M vs. IMP_A, and LDR_M vs. LDR_A groups, GO enrichment analysis was performed. Based on the results, different subspecies only had some genes related to basic metabolism during the improvement (Supplementary Figure S1C). However, the gene frequency was high in *ssp.* compared with that in *ssp. agrestis*, and Melo was more enriched in GO items related to abiotic stress responses and the synthesis of flavonoids (Figure 2C). Flavonoids have been proven to play an important role in abiotic stress (Treutter, 2006).

### Genome-Wide Distribution of RGA Candidates

**Figure 3:**
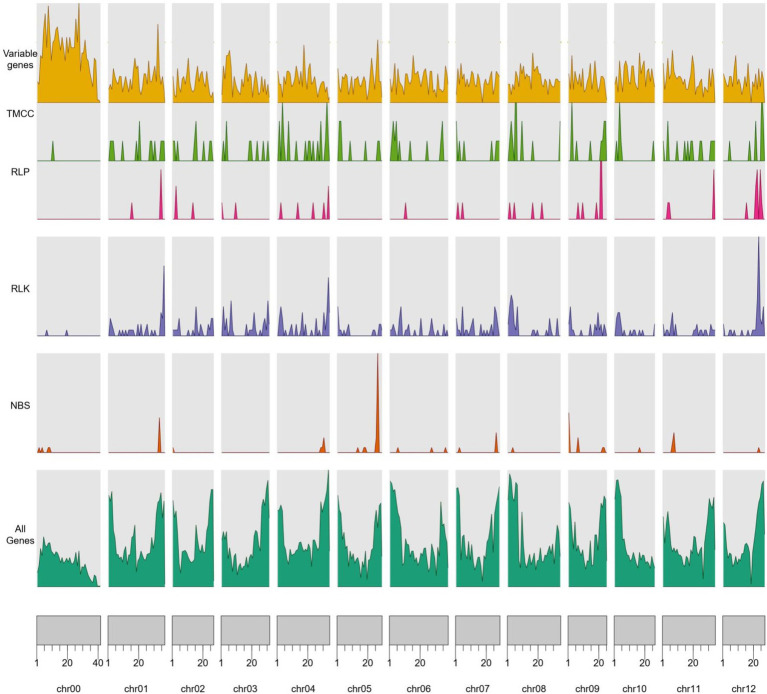
The distribution of variable genes and nucleotide binding site (NBS), receptor-like protein (RLP), receptor-like protein kinase (RLK), and transmembrane coiled-coil domain protein (TM-CC) domains across the reference genomes.

A total of 709 RGAs were identified in the *Cucumis melo pan*-genome (Figure 3; Table 1). RLK was the largest candidate resistance gene group and contained 411 genes; this was followed by TM-CC, which contained 141 candidates (Table 1). Among the 709 RGA candidates, 603 (85%) were core genes (soft core and core) and 106 (15%) were variable genes (cloud and shell genes). RLKs showed the highest number of gene presence/absence variations (94.2%), followed by TM-CC (92.2%), while RLPs showed the lowest percentage of gene presence/absence variations (32.1%). The high percentage of variable genes that are incomplete (RLK and TM-CC) could mean that some of these genes may be pseudogenes that can be lost by the genome without consequences in the form of lost resistance. A total of 654 RGA candidates (603 cores and 51 variables) were identified in the reference, and 55 RGA candidates (all variables) were on pan-genomic additional contigs. The RGA candidate density per pseudomolecule completely differed (average 1.74/Mb, and each RGA candidate ranging from 0.17/Mb on chr00 to 2.42/Mb on chr04), and the density of different types of RGAs per pseudomolecule also differed (there were no NBS genes on chr03, and the density on chr05 reached 0.89; RLK genes per Mb ranging from 0.05 on chr00 to 1.56 on chr12; there was no RLP distribution on chr00, chr05, and chr10, and 0.36 per Mb was the largest density; and TM-CC density ranging from 0.02 per Mb on chr00 to 0.61 per Mb on chr04). Furthermore, RGA genes located on the reference pseudomolecule harbored more NBS-LRR, RLK, RLP, and TM-CC candidate genes (73, 400, 46, and 135, respectively) than pan-genome additional contigs (28, 11, 10, and 6, respectively).

**Table 1:** The number of different types of resistance gene analog (RGA) candidates and subfamilies found on the reference genomes and pan-genome additional contigs.

RGAs	Reference	Pan-genome additional contigs	Pan-genome
CN	3 (1, 2)	4 (4, 0)	7 (5, 2)
CNL	15 (3, 12)	5 (5, 0)	20 (8, 12)
NBS	8 (6, 2)	5 (5, 0)	13 (11, 2)
NL	21 (8,13)	8 (8, 0)	29 (16, 13)
RLK	400 (13, 387)	11 (11, 0)	411 (24, 387)
RLP	46 (8, 38)	10 (10, 0)	56 (18, 38)
TM-CC	135 (5, 130)	6 (6, 0)	141 (11, 130)
TN	1 (0, 1)	0 (0, 0)	1 (0, 1)
TNL	17 (5,12)	3 (3, 0)	20 (8, 12)
TX	5 (1, 4)	2 (2, 0)	7 (3, 4)
OTHER	3 (1,2)	1 (1, 0)	4 (2, 2)
Total	654 (51, 603)	55 (55, 0)	709 (106, 603)

The numbers in parentheses represent the number of variable genes (cloud and shell genes) and core genes (softcore and core), respectively.

In our study, an improved RGA candidate prediction pipeline was used, and 73 NBS-LRR genes were identified in the reference assembly. However, previous studies only identified 14 NBS-LRR in four regions, the number of which is markedly lower than that in this study. Further, genome-wide identification of NBS-LRR was not conducted (Van Leeuwen et al., 2005).

The number of NBS-LRR genes identified melon genome (73) is higher than watermelon (44; Xu et al., 2013), cucumber (61; Huang et al., 2009), and papaya (35; Ming et al., 2008) but is only a fraction of that in rice (600; Yu et al., 2002), *Brassica oleracea* (556; Bayer et al., 2019), and apple (575; Velasco et al., 2010).

Receptor-like protein kinase is the largest class of RGA candidates, aligning with other plants, such as wild strawberry, *B. oleracea*, and cotton (Chen et al., 2015; Li et al., 2018; Bayer et al., 2019). The number of RLKs is greater than that of NBS-LRRs and RLPs, which may be due to the diverse functions of different types of RGA candidates. RLK genes are reported to be involved in a variety of regulatory processes, such as self-incompatibility, interaction with symbionts, and regulation of growth processes in response to hormones (Morillo and Tax, 2006). Therefore, RLKs are not necessary for the resistance. However, other RGA genes, such as NBS-LRR and RLP, mainly focus on resistance (McHale et al., 2006).

**Figure 4:**
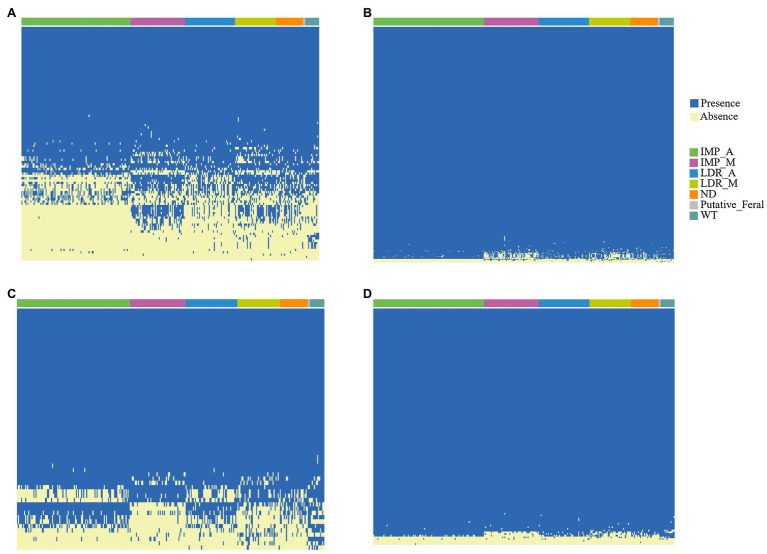
About 297 accessions heatmap revealing the presence and absence of four RGA subgroups (**A**, NBS; **B**, RLK; **C**, RLP; and **D**, TM-CC) of variable PAVs.

We compared the counts of RGA candidates within the different accessions based on the presence/absence results (Figures 4A-D). Among all subgroups of RGAs, the least proportion (1.5%) of RLK genes showed PAVs. On the contrary, the proportion (35.3%) of NBS gene with PAV was the largest. PAV analysis revealed that some RGA candidates exist only in a few varieties. For example, MEL-novelgene-2503, an NBS candidate, only exists in m4-88. The frequencies of MELO3C023577.2 in IMP_M and LDR_M were 65 and 63%, respectively, which were markedly lower than the two main subgroups IMP_A (100%) and LDR_A (100%). In contrast, the frequencies of MEL-novelgene-3583 and MELO3C031543.2 in IMP_M and LDR_M were relatively high (80, 70, 94, and 88%, respectively) compared with the other two main subgroups (0 and 22%, 19, and 48% in IMP_A and LDR_A, respectively; Figure 4A). Among RLPs, pan-genome additional gene MEL-novelgene-2206 has the lowest present frequency (0, 0, 0.07, 0.04, and 0.28 in IMP_M, IMP_A, LDR_M, LDR_A, and WT, respectively; Figure 4C).

### Gene PAV-Based GWAS

**Figure 5:**
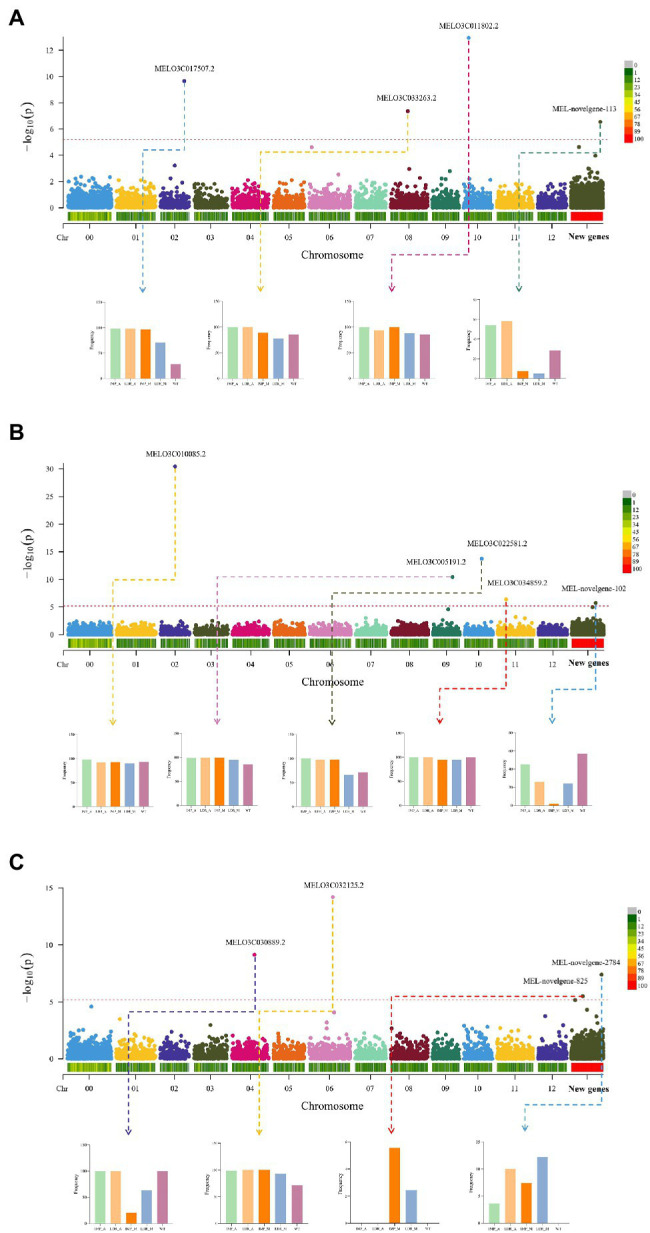
Gene PAV-based genome-wide association analysis (GWAS) of **(A)** fruit length, **(B)** fruit shape, and **(C)** fruit width. The histogram represents the frequency of genes in different groups.

In a study by Liu et al. (2020), SNP-based GWAS was performed and MELO3C035640.2.1 (CmCLV3) was found to have pleiotropic effects on carpel number and fruit shape. PAV is a major class of genome structure variation (SV). The PAV gene can also cause phenotypic changes. In the present study, 7,788 shell genes were used to conduct the gene PAV-based GWAS analysis to assess the genes located in the reference genome and additional pan-genome contigs. We identified four genes associated with fruit length, one (MEL-novelgene-113) of which was present on additional pan-genome contigs. As shown in Figure 5A, MEL-novelgene-113 had low gene occurrence frequencies in IMP_M and LDR_M. Meanwhile, the fruit length-associated gene, MEL03C017507.2, was absent in most of the accession genomes of WT melon. Herein, four reference genes and one additional pan-genome gene were found to be associated with fruit shape. As shown in Figure 5B, the additional pan-genome gene, MEL-novogene-102, had a low occurrence frequency in the IMP_M group. In the PAV-based GWAS results of fruit width (Figure 5C), two *de novo*-assembled genes (MEL-novelgene-2784 and MEL-novelgene-825) were identified as significant signals. MEL-novelgene-825 was absent in IMP_A, LDR_A, and WT groups, and only existed in a few accessions.

### PAV of the *TPS* Gene Family

**Figure 6:**
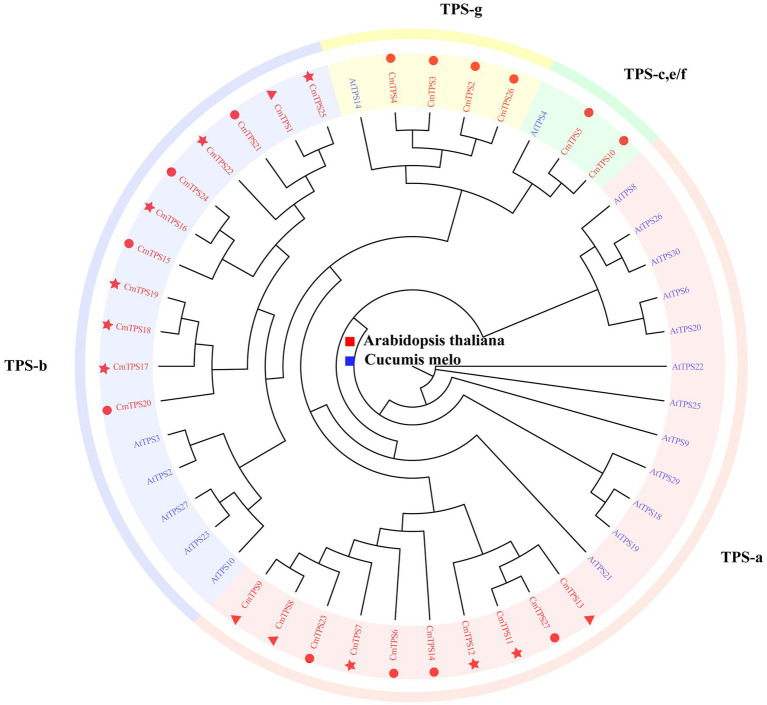
Phylogenetic tree of TPSs from melon and *Arabidopsis*. *CmTPSs* are colored red. AtTPSs are colored blue. The triangle, round, and star in front of the *CmTPSs*, respectively, represent softcore, core, and shell genes. The phylogenetic tree was constructed using the neighbor-joining (NJ) method and the Jones–Taylor–Thornton (JTT) model with 1,000 bootstrap replicates in FastTree v2.1.11.

Terpenoids have a special aroma and volatility and play an important role in regulating various biological processes in plants, especially pathogens and herbivore defense mechanisms. TPSs are key enzymes in the synthesis of terpenoids. To explore the function of the TPS gene family in the melon pan-genome, we conducted genome-wide identification of TPSs. A total of 27 TPSs were identified in the melon pan-genome, and one TPS was identified in the pan-genome additional contigs (Table 2). Most TPSs are located on chr11 (11), followed by chr06 (8) and chr12 (4). The number of TPSs located on chr00, chr01, and chr05 was the lowest, with only one TPS per chromosome. A total of 27 TPSs were classified into four groups based on the phylogenetic tree (Figure 6). CmTPSs in TPS-g and TPS-e/f were all core genes, indicating that CmTPSs in these two groups were more stable. The number of CmTPSs belonging to shell genes in TPS-b was the highest, showing that genes in TPS-b were more variable.

**Table 2:** The number of Terpene synthase (TPS) found on the reference genomes and pan-genome additional contigs.

	Reference	Pan-genome additional contigs	Pan-genome
TPS	26 (8, 18)	1 (1, 0)	27 (9, 18)

The numbers in parentheses represent the number of variable genes (cloud and shell genes) and core genes (softcore and core), respectively.

We linked the TPSs with the PAV selection analysis. CmTPS27 is an unfavorable gene in the group of IMP_M vs. LDR_M, and the frequency was significantly higher in LDR_M in the group of LDR_M vs. LDR_A, revealing that CmTPS27 suffers negative selection during the process of improvement, and may be related to special traits in *ssp. Melo*. The frequency of CmTPS12 was significantly higher in the *ssp. Melo* in the group of LDR_M vs. LDR_A and was a favorable gene in the IMP_M vs. IMP_A group, which showed that CmTPS12 may be related to special traits in *ssp. Melo* and under positive selection pressure during the process of improvement The frequency of CmTPS16, CmTPS17, CmTPS18, and CmTPS25 was significantly lower in LDR_M, indicating that these TPSs may be related to special traits in *ssp. agrestis*.

## Discussion

During domestication and crop improvement, many genomic variations will appear. SNP is a type of variation that has been frequently studied for more than 10 years. Many studies have shown that structural variation plays an important role in regulating plant traits. Gene PAV is a type of structural variation, and pan-genome construction is necessary for gene PAV calling. In this study, we constructed a pan-genome of melon, which contains 168 Mb novel sequences and 4,325 novel genes. The pan-genome contains more complete genome information of the species, including the absence of core genes and genes in some individuals. However, the size of the pan-genome does not increase infinitely as the sample size increases. For example, in a pan-genome study of tomato, when the number of samples reached 300, the growth of pan-genome became very small (Gao et al., 2019).

Crop improvement usually focuses on some main agronomic traits and ignores some other traits. The improvement of tomato usually ignores organoleptic/aroma quality traits in the process of improvement, which results in the loss of some genes in this process (Gao et al., 2019). The gene PAV differences between different groups can be due to genetic drift, they could be random or due to respective positive or negative selection. Due to the PAV gene, many genes in the reference genome, DHL92 V3.5.1, are missing in other melon individuals. To comprehensively identify the resistance gene resources of melon, we used the pan-genome of melon to identify RGA and analyzed its distribution, presence/absence, and gene PAV selection among populations. Similar to the pan-genome research of *Brassica oleracea* and *Brassica napus* (Bayer et al., 2019; Dolatabadian et al., 2020), our study provides more abundant resources for melon resistance research. Similar to other plants, the distribution of RGA in the melon genome was not uniform (Bayer et al., 2019; Dolatabadian et al., 2020), which may be due to events, such as segmental duplication.

Pan-genome studies of pigeon pea, *Brassica napus*, and chicken revealed that GWAS analysis based on PAV can identify candidate genes that differ from those identified *via* GWAS analysis based on SNPs (Song et al., 2020; Zhao et al., 2020; Wang et al., 2021). The number of PAV genes is markedly smaller than that of SNPs, but can be used to directly study the potential effect of the absence of genes on phenotype. The candidate genes identified in this study are not only located in the reference genome of melon, but are also located in additional pan-genome contigs. The annotation of MEL-novelgene-102 in COG, UniProt, and other databases shows that it is not only a type of leucine-rich repeat (LRR) protein, but also an MDIS1-interacting receptor-like kinase 2 gene. As shown in a study of *Arabidopsis thaliana*, it can connect cell wall integrity sensing, root growth, and response to abiotic and biotic stress (Van der Does et al., 2017). The absence of these proteins in IPM_M is very large, and their occurrence frequency in the wild type is very high. The relationship between this phenomenon and fruit shape regulation requires further study. Interestingly, MEL-novelgene-113 and MEL-novelgene-102 encode the same protein, indicating that they may be involved in the regulation of fruit length. Meanwhile, MEL03C017507.2, located on the reference genome, encodes the glutamate receptor 2.7-like protein, a protein that controls the defense of pathogens, reproduction, stomatal aperture, and light signal transduction in plants (Cho et al., 2009; Michard et al., 2011; Forde and Roberts, 2014). The frequency of its existence in the wild type is very low, and the occurrence frequency is higher in the local and improved types. In a study on papaya, a fruit length QTL region was found, and a gene that encodes glutamate receptor 3 was located in the region (Nantawan et al., 2019). Therefore, the regulation of melon fruit length by glutamate receptors is worthy of a further study. The fruit width-related significant genes located in the reference and additional pan-genome contigs were uncharacterized proteins, and their function requires future research. Although PAV-GWAS can get fewer significant associations than traditional SNP-GWAS, but it will provide a valuable complement to SNP-based GWAS for identifying gene present/absent variants of plant important traits.

Terpenoids play an important role in plant flavor, attracting pollinators, and defense (Qiao et al., 2021). They are mainly catalyzed by terpene synthase (TPS). Terpenoids are important components of plant volatile mixtures induced by herbivorous insects, which can attract natural enemies of herbivorous insects (He et al., 2022). A cucumber study identified the participation and function of *TPS* genes in cucumber response to herbivorous insects with different feeding habits (He et al., 2022). Several transcripts of *CsTPS* genes are upregulated in the leaves of herbivorous insects, and the products of these expressed proteins are consistent with the known terpenoids in the volatile mixtures released by herbivorous insects. Many grass TPSs play an important role in the biosynthesis of volatiles induced by herbivores, such as *TPS1*, *TPS10*, and *TPS23* in maize (Schnee et al., 2002, 2006; Köllner et al., 2008), and Os02g02930, Os08g07100 (TPS46), TPS3, and Os08g04500 in rice (Cheng et al., 2007; Yuan et al., 2008). There were 27 TPS genes identified in the melon pan-genome, which provide a basis for the study of the *TPS* genes in melon. In particular, if we combined the volatile collection in different melon accessions and the PAVs of *TPS* genes in the future study, it will provide a new insight for the insect resistance study of melon. On the whole, these results are useful for future biological discovery and breeding.

## Data Availability Statement

Publicly available datasets were analyzed in this study. This data can be found online at: https://figshare.com/articles/dataset/melon_pangenome/17195072.

## Author Contributions

YS, X-DK, and Y-JZ conceived and designed the experiments. YS, YL, JW, XW, P-TZ, and BJ contributed to paper writing. YS, W-HX, Y-QW, YL, and JW conducted the experiment. X-DK and YS contributed to the data analysis. All authors contributed to the article and approved the submitted version.

## Funding

This work was supported by the National Natural Science Foundation of China (31871964, 31401753, and 32100352).

## Conflict of Interest

X-DK is employed by Jiguang Gene Biotechnology Co., Ltd.

The remaining authors declare that the research was conducted in the absence of any commercial or financial relationships that could be construed as a potential conflict of interest.

## Publisher’s Note

All claims expressed in this article are solely those of the authors and do not necessarily represent those of their affiliated organizations, or those of the publisher, the editors and the reviewers. Any product that may be evaluated in this article, or claim that may be made by its manufacturer, is not guaranteed or endorsed by the publisher.

## Footnotes

^1^ http://www.ncbi.nlm.nih.gov/Traces/sra/sra.cgi?view=toolkit_doc&f=fastq-dump

^2^ http://cucurbitgenomics.org

^3^ https://www.arabidopsis.org/

^4^ https://github.com/rambaut/figtree/

## Supplementary Material

Supplementary Figure S1 The difference in the frequencies and GO enrichment terms of the selected genes. **(A)** Line plots of the group of IMP_M vs. IMP_A and LDR_M vs. LDR_A. **(B)** GO enrichment terms in the selected genes of IMP_A vs. LDR_A. C. GO enrichment terms in favorable (right) and unfavorable (left) genes of IMP_M vs. LDR_M.

Supplementary Figure S2 About 297 accessions heatmap revealing the presence and absence of variable PAVs.

Supplementary Table 1 The information of RNA-seq data used in pan-genome annotation.
