# Supplementary figures and images for: Pan-Genome Analysis Reveals the Abundant Gene Presence/Absence Variations Among Different Varieties of Melon and Their Influence on Traits
Source: Front Plant Sci. 2022 Mar 25;13:835496. doi: 10.3389/fpls.2022.835496 (PMC8990847; doi:10.3389/fpls.2022.835496)

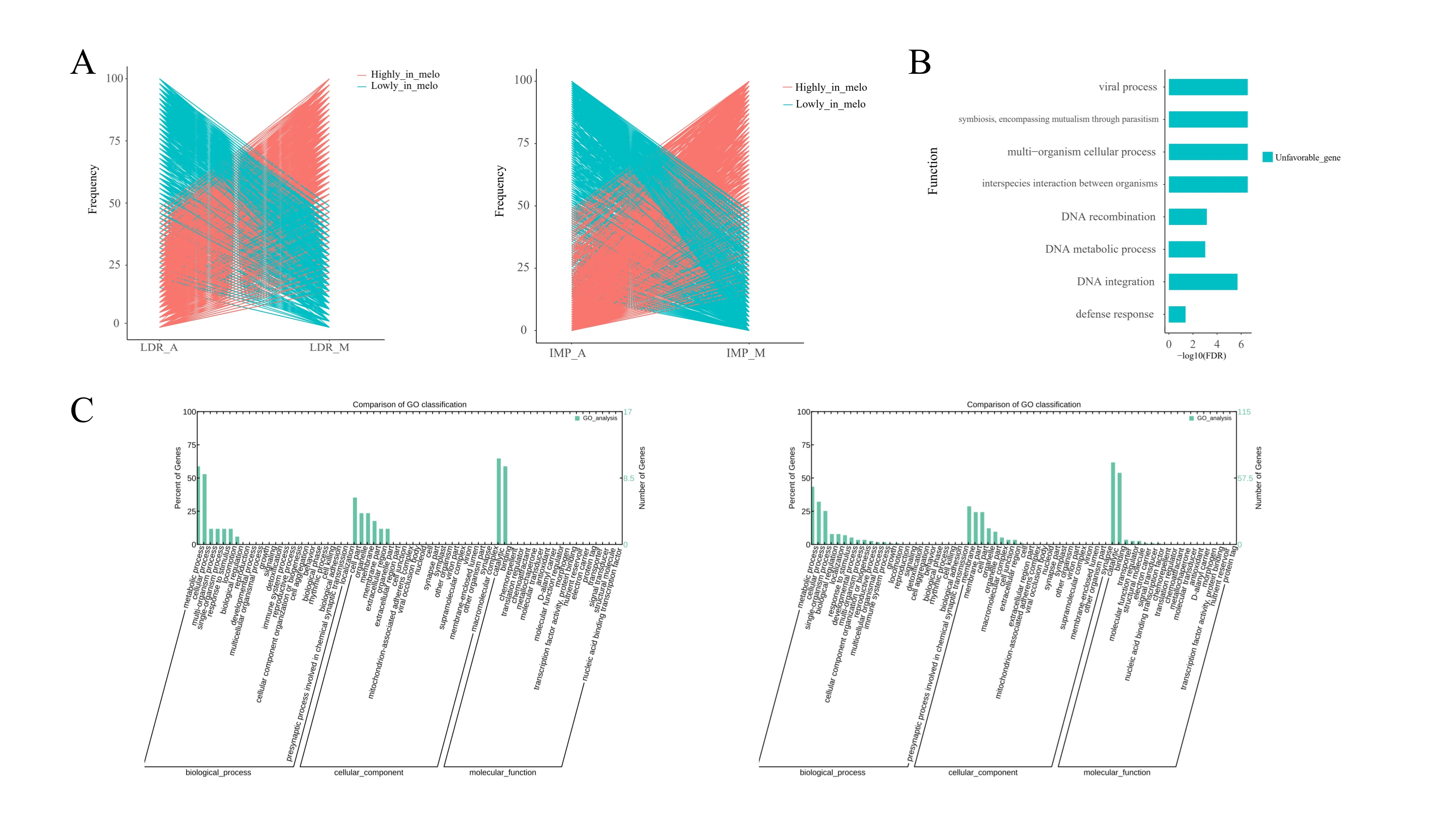

Supplement: Supplementary Figure S1 — The difference in the frequencies and GO enrichment terms of the selected genes. (A) Line plots of the group of IMP_M vs. IMP_A and LDR_M vs. LDR_A. (B) GO enrichment terms in the selected genes of IMP_A vs. LDR_A. C. GO enrichment terms in favorable (right) and unfavorable (left) genes of IMP_M vs. LDR_M. [file Image_1.JPEG]

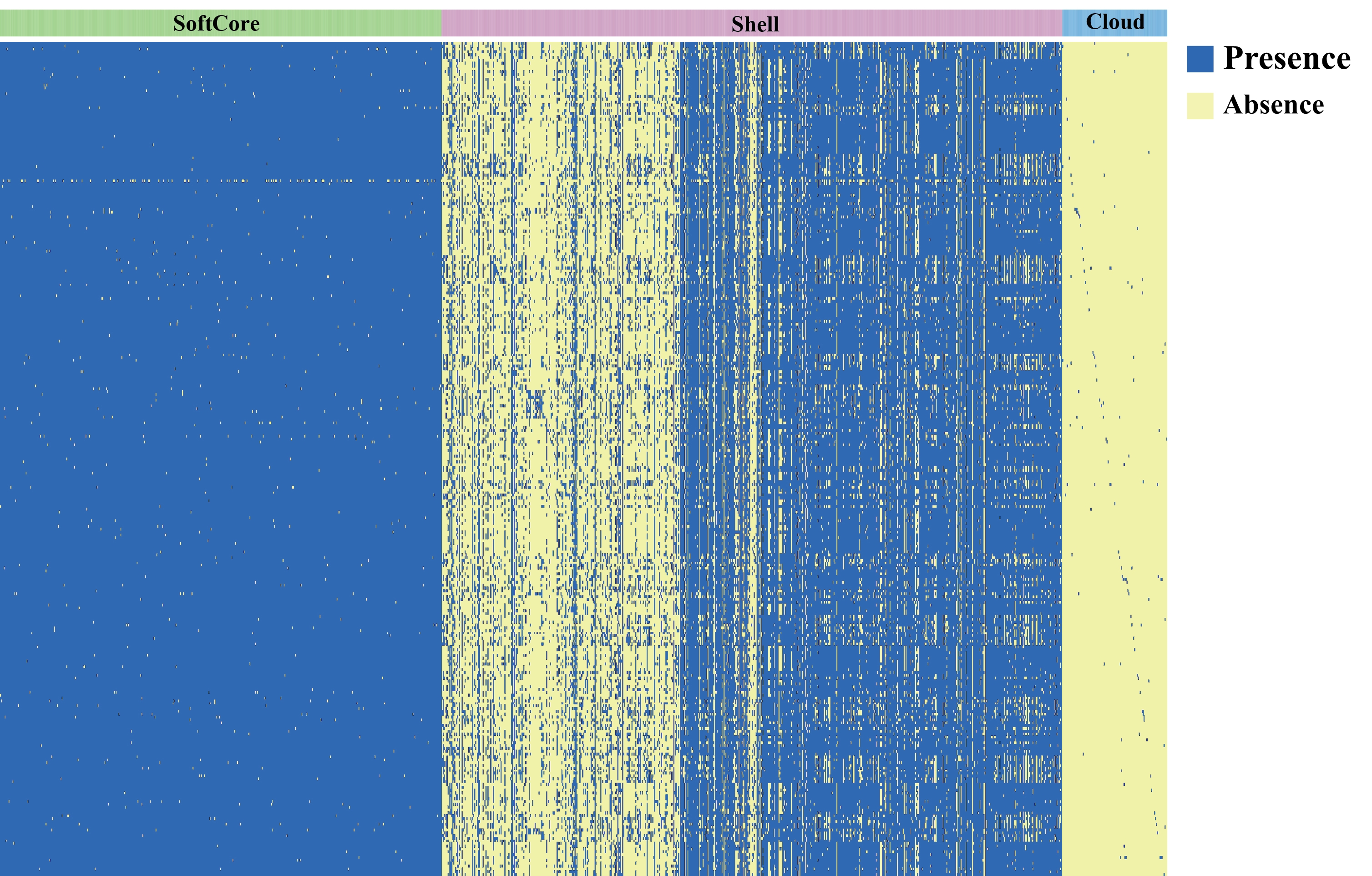

Supplement: Supplementary Figure S2 — About 297 accessions heatmap revealing the presence and absence of variable PAVs. [file Image_2.jpg]
